# Rheo-Impedance Measurements of Lamellar–Vesicular Phase-Transition Behavior

**DOI:** 10.1021/acsomega.5c07863

**Published:** 2026-01-16

**Authors:** Isao Shitanda, Ryo Kotsubo, Chihiro Hashiba, Noya Loew, Yoshifumi Yamagata, Keisuke Miyamoto, Taku Ogura, Hikari Watanabe, Masayuki Itagaki

**Affiliations:** † 13258 Tokyo University of Science, 2641, Yamazaki, Noda, Chiba 278-8510, Japan; ‡ Anton Paar Japan K. K., Riverside Sumida 1st Fl, 1-19-9, Tsutsumi-dori, Sumida-ku, Tokyo 131-0034, Japan; § Research Institute for Science and Technology, 13258 Tokyo University of Science, 2641 Yamazaki, Noda, Chiba 278-8510, Japan

## Abstract

In this study, we investigated the lamellar-to-vesicular phase transition of nonionic surfactants (BL-4.2 and BL-4SY) in concentrated aqueous solutions under shear flow using a newly developed rheo-impedance technique. Although conventional methods such as small-angle light scattering (SALS) have clarified macroscopic structural changes, the internal electrical properties during these transitions remain largely unexplored. Briefly, we simultaneously measured the viscosity and electrochemical impedance during shear-induced phase transitions and compared the results to SALS observations. For BL-4.2, scattering images revealed transitions from lamellar structures to vesicles, followed by structural collapse. In contrast, BL-4SY exhibited stable vesicle formation without collapse, likely because of its uniform ethylene oxide chain length. Further, rheo-impedance measurements showed a consistent decrease in resistance from approximately 1050 to 520 Ω during vesicle formation, and there was a greater decrease as the electrolyte concentration increased. The viscosity increased from ≈0.6 to 1.5 Pa·s, corresponding to the lamellar-to-vesicular transition, as confirmed by SALS. Interestingly, at low Na_2_SO_4_ concentrations (10^–3^–10^–2^ M), the resistance was 20–30% higher than that of the electrolyte-free sample, suggesting partial ion-trapping by sulfate ions at the surfactant termini, a phenomenon not observed for KCl. These findings demonstrate that rheo-impedance analysis can characterize both the structural evolution and ionic transport during surfactant phase transitions, offering new insights for the design and evaluation of dispersions and drug delivery systems.

## Graphical abstract

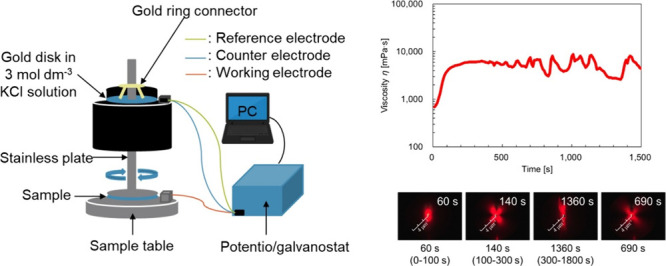

## 1 Introduction

Multilayer vesicles formed by the application of shear to lamellar structures comprising amphiphilic molecules have attracted interest since Bangham and co-workers first reported the bilayer structures of phospholipids.^1^ Vesicles composed of biomolecules such as phospholipids are promising drug carriers because of their high biocompatibility.^2–5^ Following Kunitake’s discovery that vesicles can also form from surfactants,^6–8^ various synthetic cationic surfactants and mixtures have been studied.^9–17^

Vesicles have been utilized in household products,^12–14^ foods,^15,16^ and cosmetics,^17,18^ and those composed of nonionic surfactants (niosomes; vesicular assemblies formed from nonionic surfactants, analogous to liposomes but composed of synthetic amphiphiles.)^19–23^ are particularly attractive for their high stability and facile molecular design. In addition, their tunable hydrophilic and hydrophobic structures allow precise control of bilayer properties, making them versatile models for investigating shear-induced self-assembly. C_12_E_*m*_, a typical nonionic surfactant, self-assembles in aqueous solution above its critical micelle concentration (CMC). Further, surfactants having *m* values between 3 and 6 form lamellar phases across broad temperature and concentration ranges. When shear is applied under specific thermal conditions, niosomes readily form.^24–30^ This enables the straightforward preparation of drug delivery system (DDS) carriers by simply incorporating an active ingredient, such as a drug, before shear application, followed by shear-induced mixing.

Recently, the lamellar-to-vesicular phase transition of an inexpensive industrial-grade surfactant, C_12_E_4.2_ (a nonionic surfactant containing ethylene oxide (EO) units), was confirmed using small-angle X-ray scattering (SAXS) and small-angle light scattering (SALS) measurements. The system comprised 40 wt % C_12_E_4.2_ and 60 wt % pure water.^31^ Analysis revealed that the interlamellar spacing increased as the temperature increased to 30 °C and that bilayer fluctuations intensified with further increase in temperature. Rheo-SALS measurements also indicated a temperature-dependent increase in viscosity, accompanied by the appearance of a clover-shaped scattering pattern, which is consistent with earlier reports. Collectively, these studies have established the macroscopic structural evolution of surfactant systems under shear, providing an essential foundation for the present investigation.

To date, previous studies have mainly focused on the macroscopic structural evolution of surfactant systems during shear-induced lamellar–vesicular transitions using rheological, SAXS, or SALS techniques.^24–31^ However, the accompanying changes in electrical properties, particularly how ion conduction evolves in response to structural rearrangement, have not been experimentally clarified.

In this study, we employed rheo-impedance spectroscopy, which enables the simultaneous and time-resolved monitoring of viscosity and electrochemical impedance under identical shear conditions.^32–34^ This approach provides a direct correlation between mechanical deformation and electrical transport during the transition process, offering quantitative insights into the coupling between the morphology and ion mobility. Such a correlation analysis has not been reported for nonionic surfactant systems to date, demonstrating the novelty of this study.

Further, this technique clarifies the conduction mechanisms within a sample by measuring electrochemical impedance during shear. Rheo-impedance has also been applied to investigate the structural evolution of soft materials under shear stress. Previous studies have demonstrated its effectiveness in characterizing the gelation dynamics of gelatin solutions and the rheological behavior of mechanically processed cellulose nanofibers.^35,36^ However, these measurements have not yet been applied to nonionic surfactant systems. By elucidating how electrochemical impedance correlates with macroscopic structural changes, we can obtain deeper insight into the mechanisms underlying shear-induced phase transitions.

Therefore, we conducted real-time impedance measurements during shear-induced lamellar–vesicle phase transitions using both industrial-grade and high-purity C_*n*_E_*m*_ surfactants. These measurements were combined with SALS to verify the correlation between the impedance spectra and time-dependent structural changes during the transition process. Importantly, this approach does not replace conventional structural probes but rather complements them by providing access to electrical transport behavior that cannot be obtained from scattering or optical techniques alone.

## 2 Experimental Section

### 2.1 Rheo-SALS Measurement

Rheo-SALS measurements^31,37,38^ were conducted to correlate the viscosity behavior of a surfactant-concentrated aqueous solution during phase transitions with the corresponding structural changes observed in the scattering images. Figure 1 shows the apparatus used for the measurements. A rheometer (Anton Paar, MCR 102) equipped with a Rheo-SALS accessory was used. The nonionic surfactants used were BL-4.2 (NIKKOL BL-4.2, Nikko Chemicals), an industrial-grade product, and BL-4SY (NIKKOL BL-4SY, Nikko Chemicals), a high-purity version. A 40 wt % surfactant solution was prepared using pure water. We selected 40 wt % based on the phase diagram and experimental conditions reported by Hatakeyama et al.,^31^ where lamellar–vesicle transitions occur reproducibly. This concentration also provides sufficient viscosity contrast for simultaneous rheology and impedance measurements. The mixture was first heated to 40 °C and subsequently mixed at 2000 rpm for 3 min and then at 2200 rpm for 1 min. Finally, the mixture was allowed to stand at room temperature until any bubbles disappeared. Mixing was performed by using an Awatori Kneader ARV-310 (THINKY). The temperature of the apparatus was maintained at 35 °C. Each sample was placed in the apparatus and allowed to stand for 3 min to reach thermal equilibrium. The jig consisted of a 43 mm diameter quartz glass plate, and the height was adjusted to maintain a 1 mm gap between the sample and the plate. Any excess sample protruding from the jig was removed. Viscosity was measured under a constant shear rate of 10 s^–1^ throughout the entire measurement. Simultaneously, the sample was irradiated with a 658 nm laser, and the scattering images were captured using a CCD camera. For the BL-4.2 solution, images were recorded every 20 s; for the BL-4SY solution, images were recorded every 60 s. Measurements were performed over a 30 min period.

**Figure 1:**
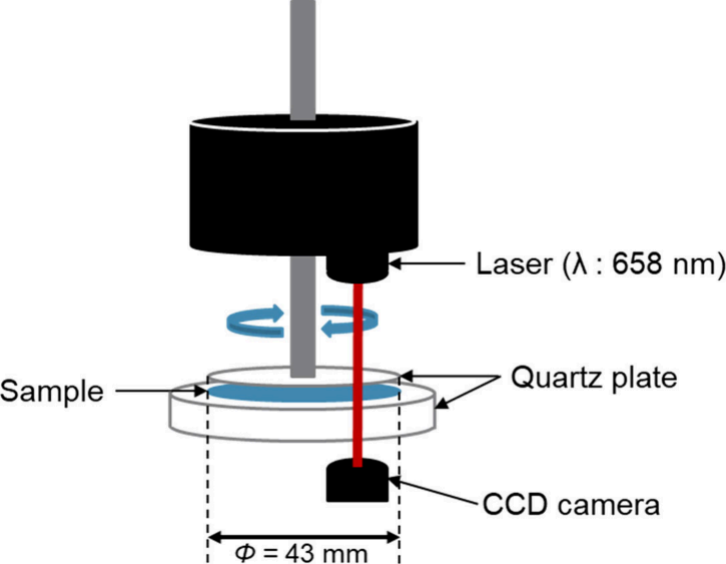
Schematic of the Rheo-SALS measurement system.

The viscosity measurements were carried out using a stainless-steel parallel-plate rheometer (Anton Paar MCR series, diameter = 50 mm, gap = 1 mm). The instrumental error of the viscosity measurement (as specified by the manufacturer) is within ±1%. To reduce potential measurement errors arising from sample handling, the sample was carefully loaded onto the lower plate to avoid preshear and was left to stand for 10 min before measurement to allow the release of residual stress. The sample amount was adjusted to fill the plate gap fully without excess material adhering to the upper plate because an insufficient sample volume can lead to artificially low viscosity values. SALS measurements were performed twice to confirm the trend.

**1:** Rheo-SALS Measurement Conditions

Temperature [°C]	35
Shear rate [s^–1^]	10
Gap [mm]	1.0
Jig	Quartz parallel-plate PP43 (**⦶** = 43 mm)
Laser wavelength [nm]	658
Shooting interval [s]	BL-4.2:20, BL-4SY: 60
Measurement time [min]	30

### 2.2 Rheo-Impedance Measurements

Rheo-impedance measurements were performed to correlate the impedance changes with the viscosity of a surfactant-concentrated aqueous solution undergoing a phase transition. Figure 2 shows the apparatus used for the measurements. A rheometer (Anton Paar, MCR 102) and a potentiogalvanostat (Meiden Hokuto Co., Ltd., HZ-7000) were employed for the experiments.

**Figure 2:**
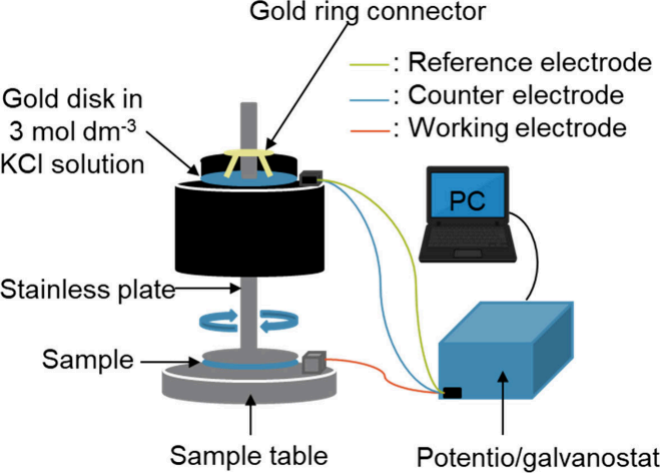
Diagram of the rheo-impedance measurement system.

Samples were prepared using electrolyte solutions (Na_2_SO_4_ and KCl) at concentrations of 1.0 × 10^–3^, 1.0 × 10^–2^, 1.0 × 10^–1^, and 1.0 M. Na_2_SO_4_ and KCl were used as supporting electrolytes, as they are commonly employed in electrochemical measurements. These two types were selected to verify the effects of differences in the ion size and charge valence. These were mixed to achieve a BL-4.2:pure water:electrolyte weight ratio of 40:55:5. The solutions were heated to 40 °C and then mixed at 2000 rpm for 3 min and further mixed at 2200 rpm for 1 min. After being mixed, the solutions were left to stand at room temperature until the foam dissipated. A control solution without an electrolyte was prepared for comparison with the rheo-SALS measurements. The BL-4SY solution was prepared by using the same procedure. The results are reported based on the electrolyte concentrations used in the preparation. A rotary mixer (Awatori Kneader ARV-310, THINKY) was used for all of the mixing steps.

The apparatus was maintained at 35 °C, and each sample was allowed to stand for 3 min to equilibrate to the set temperature. Each impedance measurement was repeated three times under identical shear conditions, and the mean values with 68.3% confidence intervals were used for analysis. The fitting residuals for the equivalent circuit were below 2%, confirming the reproducibility and reliability of the impedance data.

The stainless-steel jig had a diameter of 50 mm, matching that of the rheo-SALS setup. The gap between the sample and the plate was fixed at 1 mm, and any excess sample protruding from the jig was removed. Viscosity was measured under a constant shear rate of 10 s^–1^ throughout the entire measurement. Impedance was recorded using the potentiogalvanostat under the following conditions: initial potential 0 V, frequency range 1 to 500 kHz, and potential amplitude 10 mV. Measurements were conducted for 30 min at each electrolyte concentration. Table 2 summarizes the conditions used for the rheo-impedance measurements. In this study, we selected the high-frequency range to capture the dominant changes in solution resistance associated with structural changes. This is because, in the low-frequency region (<1 kHz), parasitic impedances from the electrode and fixture components dominate, making it impossible to observe bulk ionic responses.

**Table 2:** Rheo-Impedance Measurement Conditions

Temperature [°C]	35
Shear rate [s^–1^]	10
Gap [mm]	1.0
Jig	Stainless-steel parallel plate PP50 (**⦶** = 50 mm)
Initial potential [V]	0
Frequency range [kHz]	1–500
Amplitude [mV]	10

Note that we stirred and degassed the sample the day before measurement, let it stand overnight, visually confirmed no phase separation had occurred, and then proceeded with the measurement. The confidence intervals for the error bars in each figure are described using standard deviation. Furthermore, the confidence intervals are based on point-by-point variation values with three measurements taken for each. The overall experimental scheme is shown in Scheme S1.

## 3 Results and Discussion

### 3.1 Rheo-SALS Measurement

Figure 3 shows the time–viscosity curves and scattering images of a 40 wt % BL-4.2 solution obtained from rheo-SALS measurements. The elliptical scattering patterns indicate lamellar structures, whereas the cloverleaf-shaped scattering patterns indicate vesicular structures.^28,30,31^ Scattering images indicative of the lamellar structure were observed up to 100 s after the start of the measurement. From 100 to 300 s, the scattering images showed vesicle structures. Beyond 300 s, the images primarily exhibited disordered scattering; however, occasionally clover-shaped scattering patterns, such as those observed around 690 s, were also observed for 2–3 s. This suggests that while most of the structures in the measured region have collapsed, a small number of vesicles remained, indicating that total, simultaneous structural collapse does not occur upon shear application.

**Figure 3:**
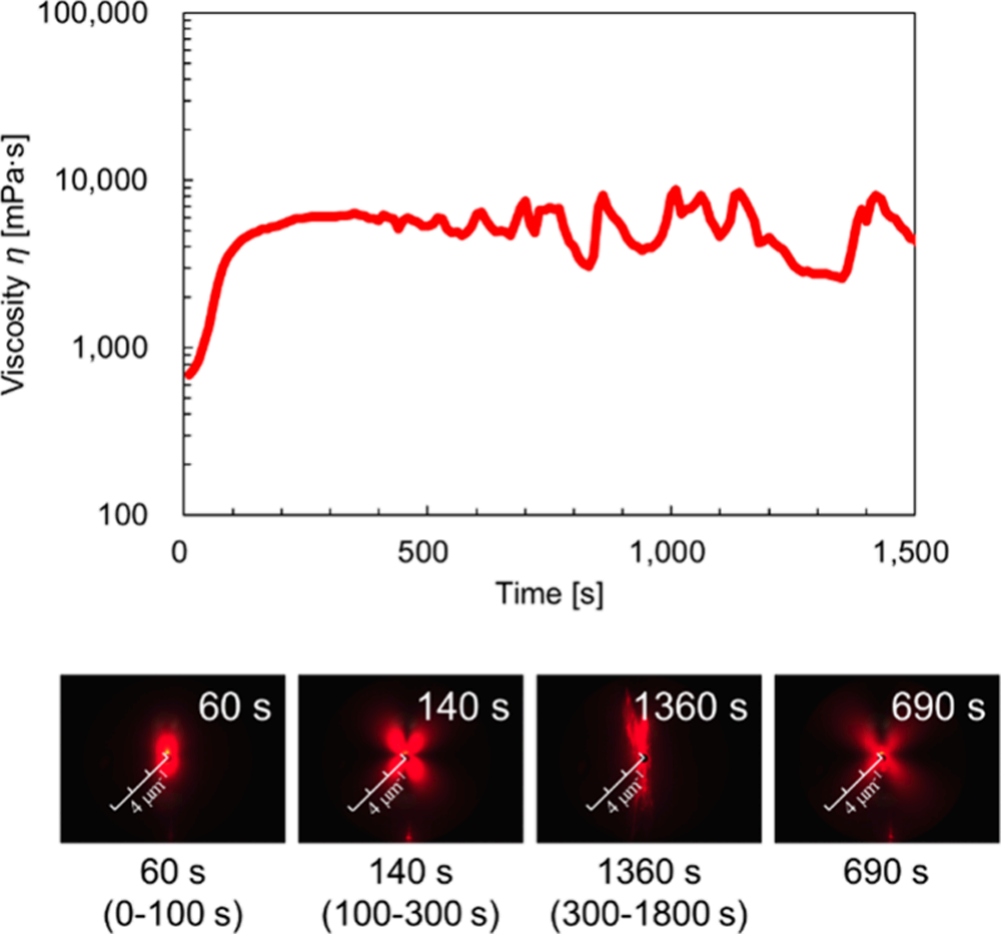
Time–viscosity curve and scattering images of the 40 wt % BL-4.2 solution obtained by rheo-SALS measurement. Scattering images correspond to specific time points and illustrate the time-dependent structural evolution under shear. The period from 100 to 300 s is that in which lamellar layers reorganize into vesicle structures, producing clover-shaped scattering patterns with only gradual viscosity changes. Beyond 300 s, disordered scattering patterns become dominant; however, occasional clover-shaped scattering patterns (e.g., around 690 s) appear, suggesting transiently remaining vesicular domains within a largely collapsed structure. These transitions correspond to the three regimes shown in the viscosity curve: rapid increase (0–100 s, lamellar), slow increase (100–300 s, vesicle formation), and fluctuation (300–1800 s, structural collapse and reorganization). Each scattering image corresponds to a specific time point, and the series of images illustrate the temporal evolution of the structure during shear application.

The viscosity remained high during this period, and the scattering images corresponding to the lamellar structure (0–100 s) coincided with a rapid increase in viscosity. During the period when vesicle structures were observed (100–300 s), the viscosity increased more slowly. In the later stage, when disordered scattering was predominant (300–1800 s), the viscosity again increased drastically.

Figure 4 shows the time–viscosity curves and scattering images of a 40 wt % BL-4SY solution obtained from rheo-SALS measurements. The scattering images indicative of the lamellar structure were observed up to 120 s after the start of the measurement. From 120 s onward, the images exhibited vesicular structures. These observations were compared to the time–viscosity curves. As with BL-4.2, the viscosity increased rapidly during the period when lamellar structures were observed (0–120 s), followed by a more gradual increase during the period corresponding to vesicular structures (120–1800 s). However, unlike BL-4.2, no disordered scattering patterns or large fluctuations in viscosity were observed.

**Figure 4:**
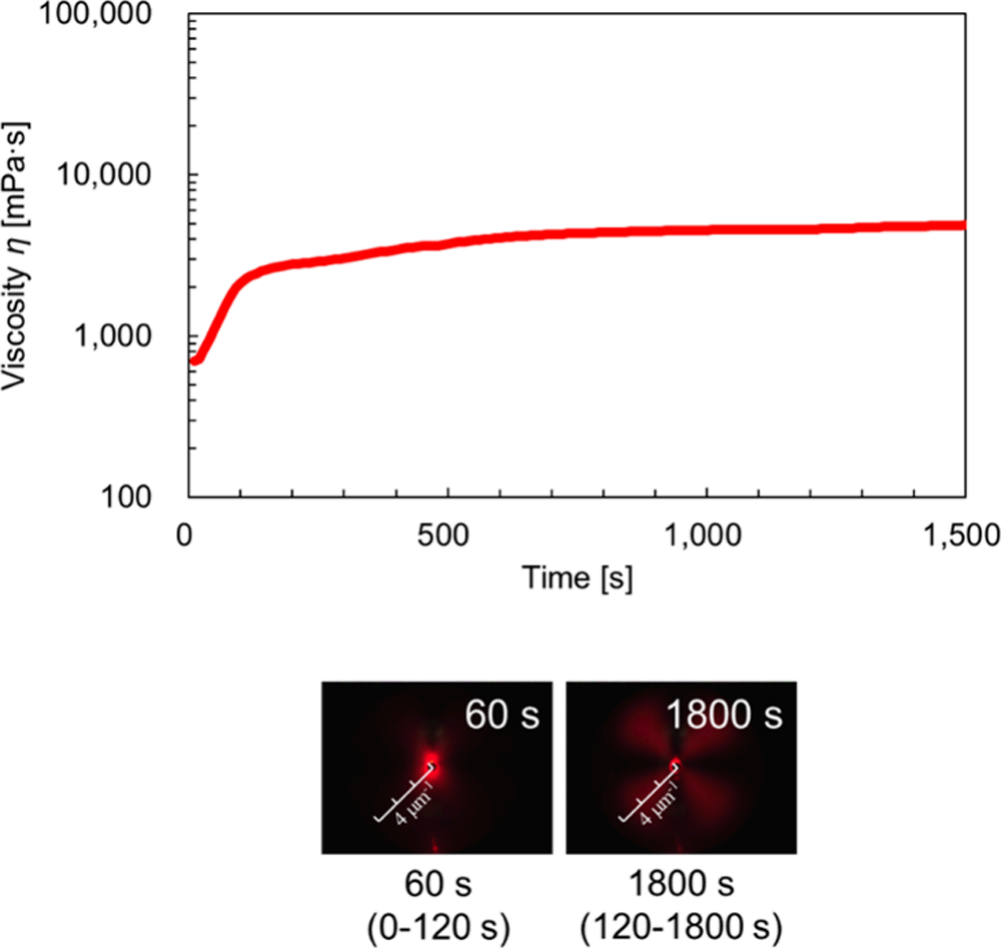
Time–viscosity curve and scattering images of the 40 wt % BL-4SY solution. Each scattering image corresponds to a specific time point, and the series of images illustrate the temporal evolution of the structure during shear.

A common feature observed in the measurements of both BL-4.2 and BL-4SY is that the initial period of rapid viscosity increase corresponds to the presence of lamellar structures, whereas the subsequent more gradual increase in viscosity corresponds to vesicle formation. However, a key difference is that for BL-4.2 the vesicle scattering images gradually deteriorate, becoming more disordered. This behavior may be attributed to ion trapping differences in the chain length between the two surfactants resulting from their different EO contents. Specifically, BL-4.2 has a distribution of EO chain lengths with an average of 4.2 units, whereas BL-4SY contains exactly four EO units per molecule. Consequently, BL-4.2 showed greater fluctuations in viscosity and rougher scattering patterns, suggesting structural heterogeneity likely arising from the distribution of EO chain lengths. In contrast, BL-4SY, which has a fixed number of EO units, showed a smoother viscosity evolution with no abrupt changes. Although quantitative evaluation of vesicle size uniformity requires detailed image analysis, the smooth changes in viscosity suggest that the BL-4SY system is more homogeneous and resistant to shear-induced structural collapse (Figure 5).

**Figure 5:**
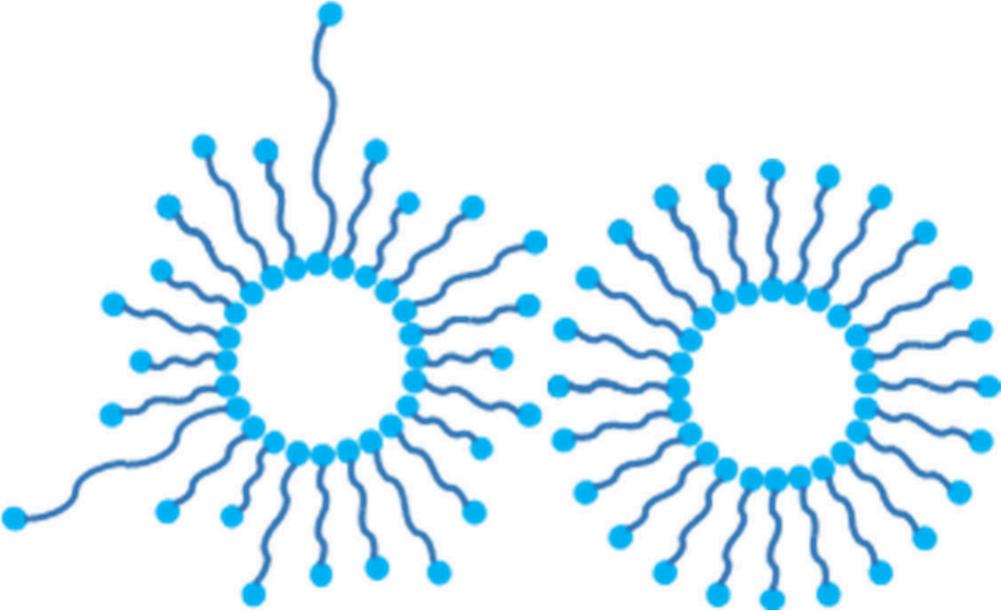
Schematics of vesicles of BL-4.2 (left) and BL-4SY (right).

### 3.2 Rheo-Impedance Measurements

Figure 6 shows the time–viscosity curve of the BL-4.2 solution containing Na_2_SO_4_. As shown in Figure 6, the viscosity behavior closely matches that observed in the rheo-SALS measurements and no distinct changes in viscosity were observed as a function of electrolyte concentration. In all cases, the lamellar-to-vesicular transition, followed by structural collapse, was clearly observed. Because we focused on the phase transition itself, impedance data corresponding to the collapse phase were excluded from the analysis.

**Figure 6:**
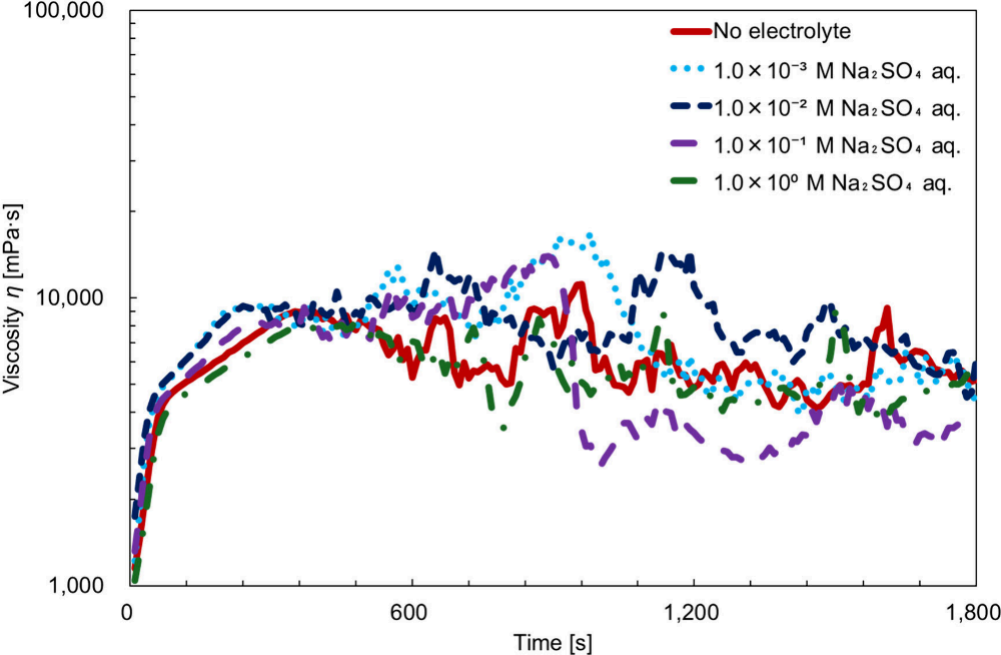
Time–viscosity curve of a BL-4.2 solution containing Na_2_SO_4_. Each sample was mixed to contain 40 wt % surfactant, 55 wt % water, and 5 wt % electrolyte. Labels in the figure indicate the concentration of the electrolyte used during mixing.

Figure 7a shows the Nyquist plots obtained from the rheo-impedance measurements of BL4.2 without electrolyte and with Na_2_SO_4_. A single depressed semicircle was observed without any additional low-frequency tail, indicating that the impedance response mainly reflects the bulk solution resistance rather than interfacial charge-transfer processes.

**Figure 7:**
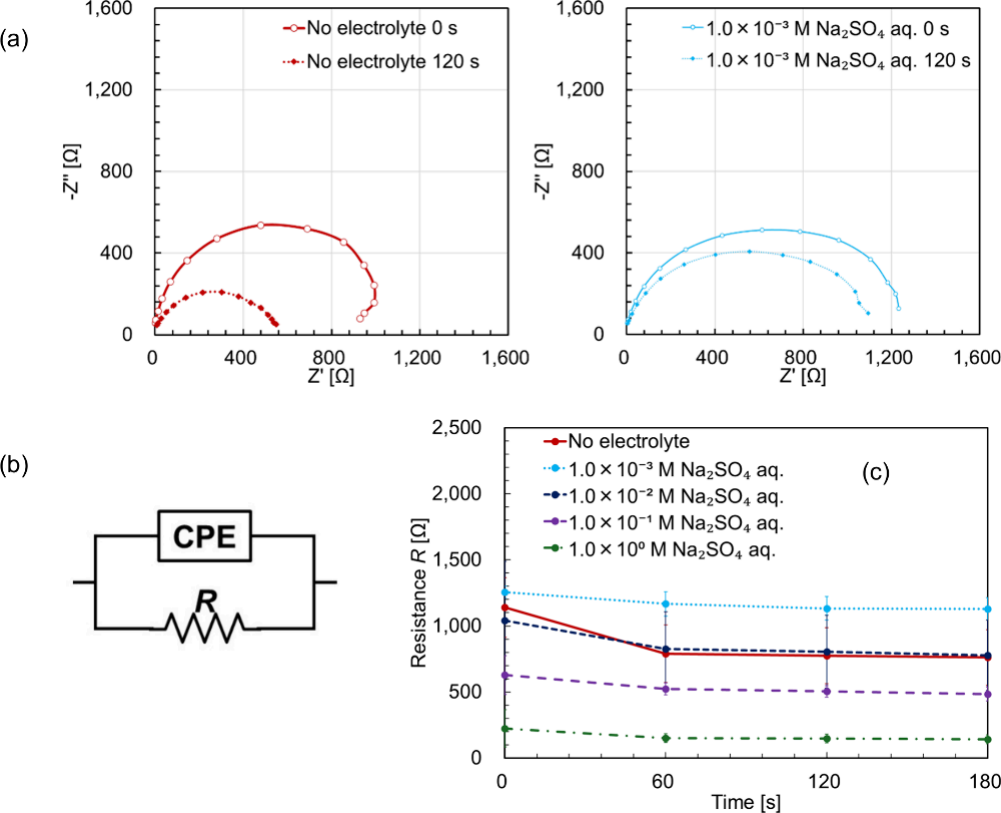
(a) Representative Nyquist plots for BL-4.2 solutions with and without Na_2_SO_4_. (b) Equivalent circuit used for fitting the impedance data, where CPE represents the constant phase element (capacitance of the solution) and *R* denotes the solution resistance. (c) Time–resistance curve of the BL-4.2 solution containing Na_2_SO_4_, showing the resistance change during the phase transition. Time–resistance curve data are mean values (*N* = 3). Error bars are displayed as 68.3% confidence intervals based on Student’s *t*-distribution. Each plot shows the average value and error bars for each data point. Each sample was mixed to contain 40 wt % surfactant, 55 wt % water, and 5 wt % electrolyte. Labels in the figure indicate the concentration of the electrolyte used during mixing.

Therefore, the spectra were analyzed using an R–CPE (constant phase element) circuit, where R represents the ionic resistance of the bulk solution and CPE accounts for the distributed capacitance arising from the heterogeneous microstructure of the lamellar and vesicular phases. The fitting was performed using *EIS*, a dedicated software developed by Meiden Hokuto Co., Ltd., with initial parameter values of *R* = 700 Ω, *p* = 1, and *T* = 10^–10^ F s^*p*–1^. This simplified model has been widely applied for dielectric and surfactant systems in which electrode reactions are absent, providing a suitable framework to describe the impedance changes associated with structural evolution under shear. In this context, the constant phase element is treated as a phenomenological descriptor reflecting distributed relaxation processes and structural heterogeneity rather than being uniquely assigned to specific microscopic features.

Figure 7c shows a decrease in resistance during the lamellar-to-vesicular phase transition. For electrolyte-containing solutions, the resistance decreased more significantly with an increase in electrolyte concentration. Interestingly, the sample containing 1.0 × 10^–3^ M Na_2_SO_4_ had higher resistance than the sample without any added electrolyte. The total fitting parameters are listed in Table S1.

Additionally, the Nyquist plot of the impedance in an electrolyte-free solution under nonshearing conditions is shown in Figure S1. The left panel shows an enlarged view of the high-frequency region, and the right panel shows the entire plot.

As shown, there are two semicircles. These are presumed to originate from the solution capacitance and resistance in the high-frequency region and from the charge-transfer resistance, electric double-layer capacitance, and parasitic impedance of the device in the low-frequency region. In contrast, when shear was applied, the semicircles corresponding to the charge transfer resistance and electric double-layer capacitance were not observed within the measured frequency range.

Figure 8a shows the time–viscosity curve of the BL-4SY solution containing Na_2_SO_4_, and Figure 8b presents the resistance curves before and after the phase transition, obtained by curve fitting using the equivalent circuit shown in Figure 7b. The 3D Nyquist plot and the corresponding instantaneous impedance spectra are shown in Figure S2. By repeated measurements at a fixed frequency, it is possible to plot semicircles along the time axis. The size of the semicircles gradually decreased because of the applied shear, eventually converging to a constant size.

**Figure 8:**
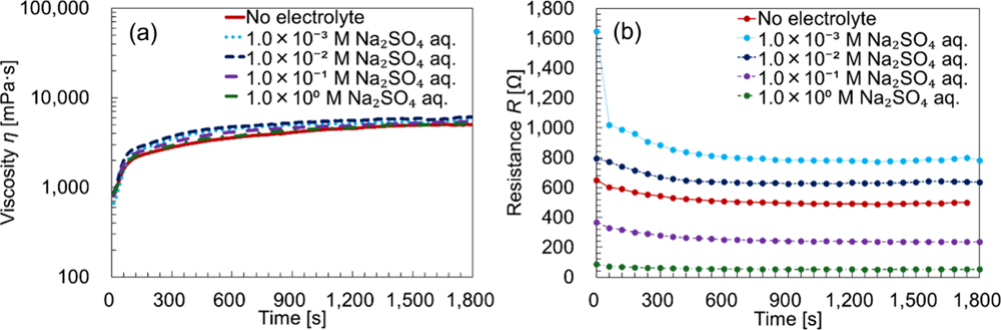
(a) Time–viscosity curve of the BL-4SY solution containing Na_2_SO_4_. (b) Resistance curves before and after the phase transition. Each sample was mixed to contain 40 wt % surfactant, 55 wt % water, and 5 wt % electrolyte. Labels in the figure indicate the concentration of the electrolyte used during mixing.

As shown in Figure 8a, the viscosity behavior closely matches that observed in the rheo-SALS measurements. No significant changes in viscosity were observed with varying electrolyte concentrations, and the lamellar-to-vesicular transition occurred consistently across all conditions.

Figure 8b shows that the resistance decreased during the lamellar-to-vesicular phase transition. In samples containing electrolytes, the resistance generally decreased with an increasing electrolyte concentration. However, the samples containing 1.0 × 10^–3^ and 1.0 × 10^–2^ M Na_2_SO_4_ displayed higher resistance than the sample without added electrolyte, suggesting a nonlinear relationship between ion concentration and conductivity during the phase transition.

To investigate the unexpected increase in resistance observed in BL-4.2 samples containing low concentrations of Na_2_SO_4_, we replaced the electrolyte with KCl and repeated the measurements. Figure 9a shows the time–viscosity curve of the BL-4.2 solution containing KCl, and Figure 9b presents the corresponding resistance curves before and after the phase transition, obtained by curve fitting using the equivalent circuit shown in Figure 7b. The highlighted regions in Figures 9a and 9b represent the same time periods. As shown in Figure 9a, the viscosity behavior was similar to that of the Na_2_SO_4_-containing samples. In addition, no significant changes in viscosity were observed with changes in KCl concentration, and the lamellar-to-vesicular transition, followed by structural collapse, occurred at all concentrations.

**Figure 9:**
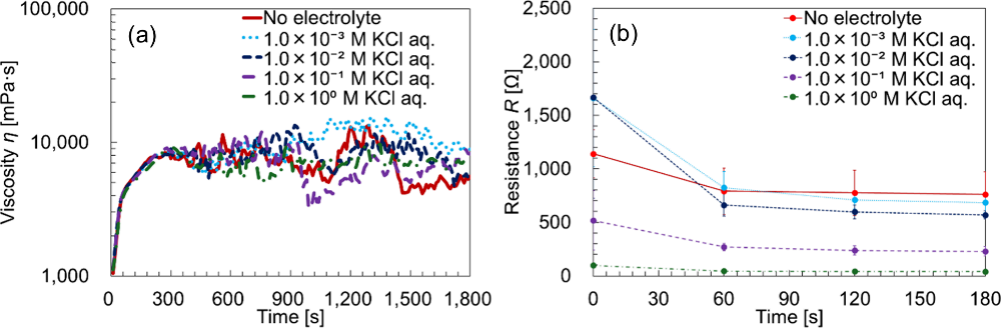
(a) Time–viscosity curve of the BL-4.2 solution containing KCl, showing the progression through lamellar, vesicular, and collapse phases. (b) Time–resistance curve of the same solution, indicating a decrease in resistance during the lamellar-to-vesicular phase transition as a function of electrolyte concentration. Time–resistance curve data are presented as mean values (*N* = 3). Each plot shows the average value and error bars for each data point. Each sample was mixed to contain 40 wt % surfactant, 55 wt % water, and 5 wt % electrolyte. Labels in the figure indicate the concentration of the electrolyte used during mixing.

Figure 9b shows that resistance decreased during the lamellar-to-vesicle phase transition. In KCl-containing solutions, resistance decreased further with an increasing electrolyte concentration. However, unlike the Na_2_SO_4_-containing samples, the resistance values of the KCl samples were no higher than those of the electrolyte-free sample. This confirms that the anomalous increase in resistance at low electrolyte concentrations is specific to Na_2_SO_4_, likely because of ion trapping or interactions with the surfactant headgroups.

Next, we consider the reason for the decrease in resistance during the phase transition. Figure 10 illustrates the lamellar and vesicular structures under shear. When shear was applied, the lamellar structure aligned parallel to the jig surface,^14^ forming a highly ordered, layered distribution. In contrast, the vesicular structures were isotropic and were uniformly dispersed throughout the sample. The corresponding Nyquist plots suggest that ions do not permeate the bilayer interfaces. In the lamellar phase, ion transport is hindered, because the stacked layers obstruct the path between the electrodes, impeding ion transport through the sample. In the vesicle phase, however, the more open and disordered structure allows ions to move more freely through the intervesicular spaces, thereby reducing the resistance.

**Figure 10:**
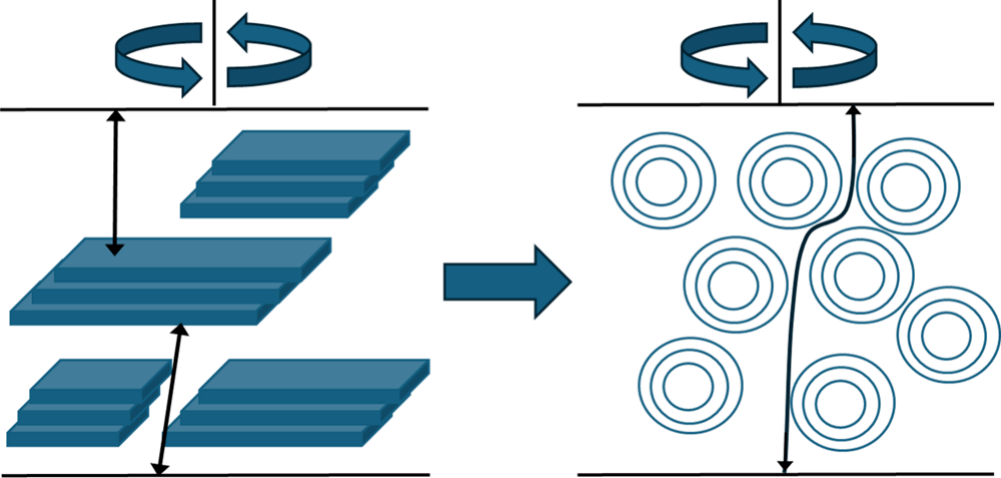
Effect of shear application on each structure.

We also considered why the sample containing a very low concentration of Na_2_SO_4_ showed a higher resistivity than the electrolyte-free sample. This phenomenon is likely due to the interaction between the sulfate anions and the terminal hydroxyl groups of the surfactant, as illustrated in Figure 11. The large, divalent sulfate ions can act as ion traps by associating with these hydrophilic termini. The resulting negatively charged sites impede the migration of cations through the system.

**Figure 11:**
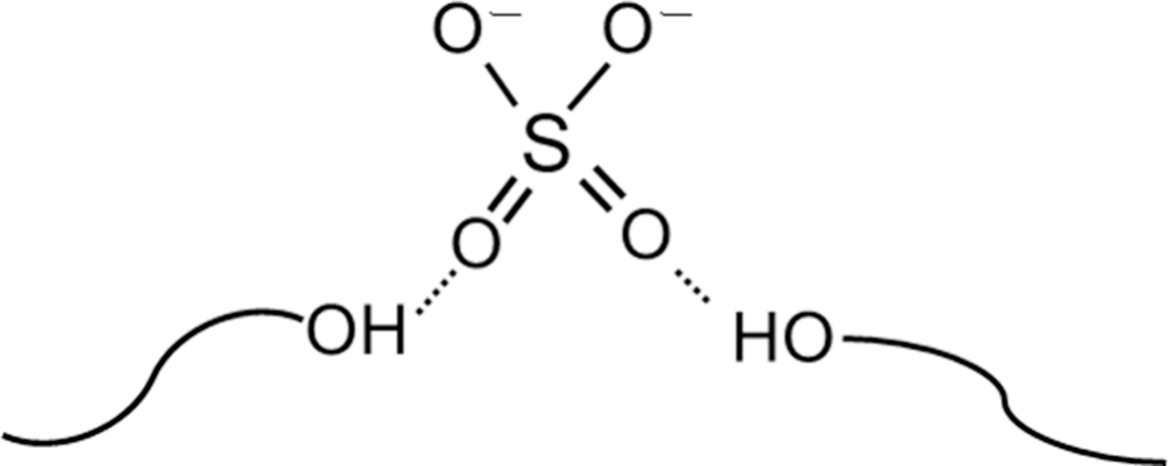
Schematic illustration of a possible ion-trapping mechanism in which sulfate ions interact with terminal hydroxyl groups of ethylene oxide chains. This figure represents a conceptual hypothesis proposed to rationalize the observed resistance increase at low Na_2_SO_4_ concentrations.

At low electrolyte concentrations, the number of free ions is limited and a significant proportion may be immobilized by these traps, leading to increased resistance. As the electrolyte concentration increased, the number of free ions exceeded the number of available trapping sites, allowing for improved ionic conduction and, consequently, lower resistance.

The above considerations remain speculative, and other mechanisms are also possible. Future research will attempt to use experimental and computational methods to verify this mechanism. Specifically,

(1) Cryo-TEM or SAXS: Direct visualization of vesicle structure, bilayer thickness, and potential multilayer structures before and after shear
(2) In situ DLS: Quantification of vesicle size changes and correlation analysis with impedance behavior during structural collapse
(3) Dielectric constant spectroscopy (ε′ and ε″): Elucidation of hydration state and local dielectric environment
(4) Molecular dynamics (MD) or double particle–particle (DPD) simulations: Providing molecular-level insights into sulfate–surfactant interactions and the origin of ion entrapment

Advancing mechanism verification through these techniques is expected to establish rheo-impedance as a valuable new tool for evaluating surfactant phase transition behavior and uncovering diverse insights.

## 4 Conclusion

In this study, the phase transition behavior of 40 wt % solutions of the nonionic surfactants BL-4.2 and BL-4SY was evaluated using rheo-impedance measurements, a novel technique for simultaneously assessing structural and electrical changes under shear. Rheo-SALS measurements revealed distinct lamellar and vesicular phases, corresponding respectively to periods of rapid and gradual increases in viscosity. In BL-4.2, the vesicular structures were unstable and collapsed over time, resulting in large viscosity fluctuations across all electrolyte concentrations. This behavior is attributed to the change in the number of EO chains in BL-4.2 (average of 4.2), unlike the uniform chain length in the purified BL-4SY (exactly four EO units). Consequently, the vesicles formed in BL-4SY remained stable over the 30 min measurement period, whereas those in BL-4.2 were disrupted by shear. The rheo-impedance measurements showed a consistent decrease in resistance following the lamellar-to-vesicular transition for both surfactants, and the resistance decreased further as the electrolyte concentration increased. An exception was observed at low concentrations of Na_2_SO_4_, where the resistance was higher than in the electrolyte-free sample. This effect was not observed with KCl, indicating that the behavior was specific to Na_2_SO_4_. The increased resistance at low Na_2_SO_4_ concentrations is likely due to ion trapping: negatively charged sulfate ions interact with terminal hydroxyl groups on the surfactant, impeding cation migration. These findings suggest that ion mobility during the phase transition is structure-dependent and influenced by the type and concentration of the electrolyte present. However, the detailed mechanisms underlying the phase transition remain unclear. Further investigation using a broader range of shear conditions and complementary measurement techniques is necessary to fully elucidate the structural dynamics fully.

Although this study demonstrates the effectiveness of the proposed impedance-based evaluation method, there are some limitations. In particular, electrode polarization may affect low-frequency impedance responses, leading to an overestimation of charge-transfer resistance under certain conditions. Future studies will focus on improving the accuracy of impedance analysis by decoupling polarization effects from intrinsic material properties.

## Supplementary Material



## Notes

3D Nyquist plots and representative Nyquist plots for BL-4SY solutions without Na_2_SO_4_ (PDF)
