# Supplementary material for: Rheo-Impedance Measurements of Lamellar–Vesicular Phase-Transition Behavior
Source: ACS Omega. 2026 Jan 16;11(5):7321–8. doi: 10.1021/acsomega.5c07863 (PMC12902838; doi:10.1021/acsomega.5c07863)
Supplement: Supplementary file 1 [file ao5c07863_si_001.pdf]

Supporting Information

Rheo-impedance Measurements of Lamellar–Vesicular Phase-Transition Behavior

Isao Shitanda[a,c,‡,], Ryo Kotsubo[a,‡], Chihiro Hashiba[a], Noya Loew[c], Yoshifumi Yamagata[b,*], Keisuke Miyamoto[b], Taku Ogura[c], Hikari Watanabe[a], and Masayuki Itagaki[a,c]*

[a] Tokyo University of Science, 2641, Yamazaki, Noda, Chiba 278-8510, Japan

[b] Anton Paar Japan K. K., Riverside Sumida 1st Fl, 1-19-9, Tsutsumi-dori, Sumida-ku, Tokyo 131-0034, Japan

[c] Research Institute for Science and Technology, Tokyo University of Science, 2641 Yamazaki, Noda, Chiba 278-8510, Japan

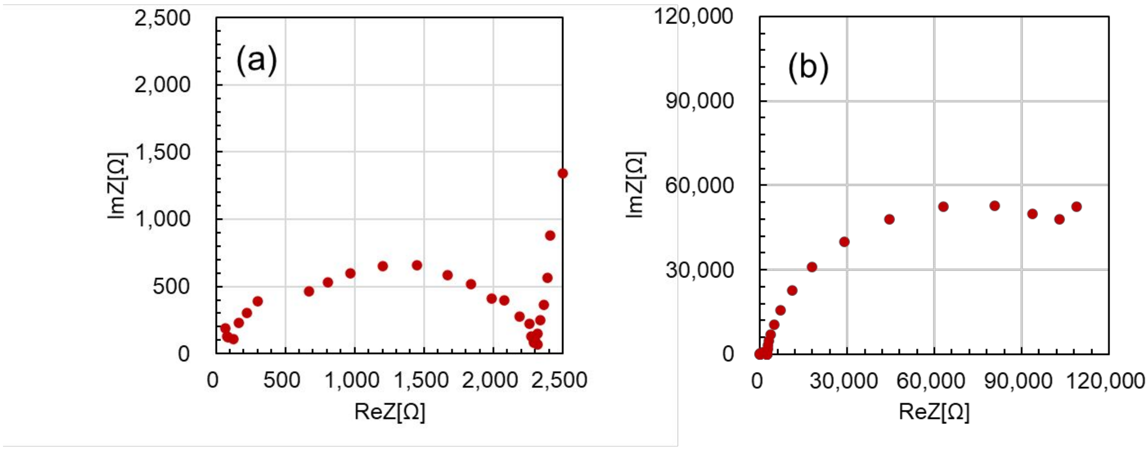

Figure S1 Representative Nyquist plots for BL-4.2 solutions without Na_2SO_4. (a) Enlarged view of (b). Measurements taken on a stationary solution in the rheometer without applied shear.

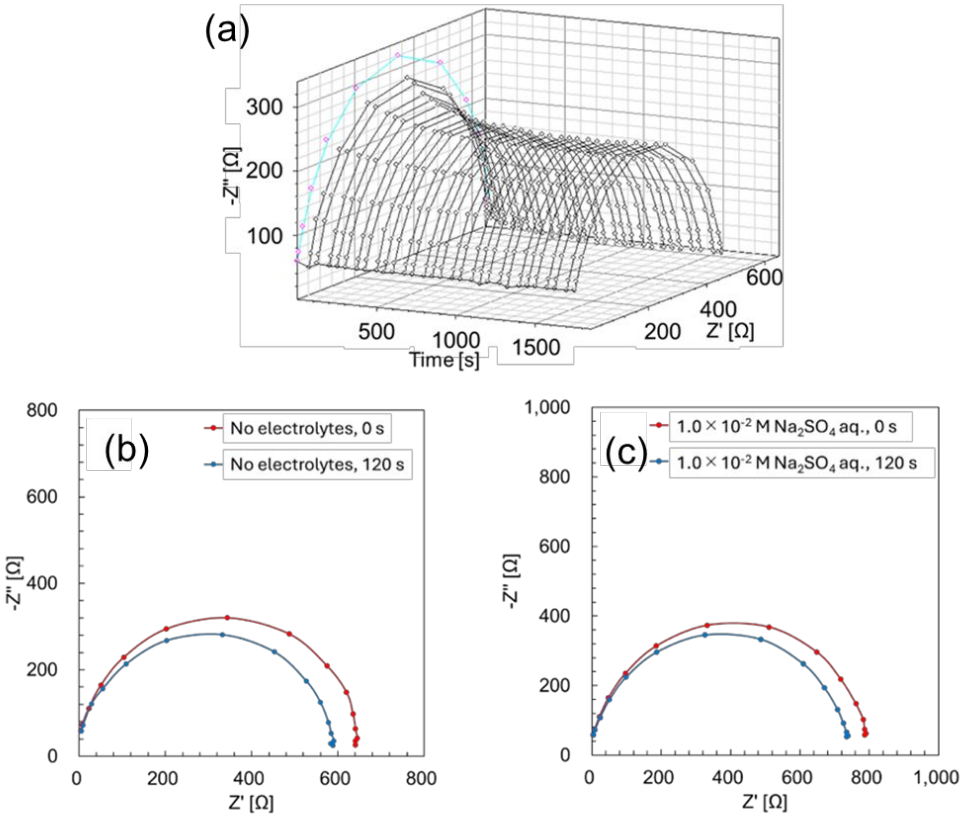

Figure S2. (a) 3D Nyquist plots for BL-4SY solutions without Na_2SO_4. By repeatedly performing measurements at a fixed frequency, we could plot semicircles along the time axis. The semicircles gradually decrease in size because of the applied shear, eventually converging to a constant size. Representative Nyquist plots for BL-4SY solutions without (b) and with (c) Na_2SO_4.

Table S1 Fitting parameters of Nyquist plots in Fig. 7c in the main text.

No electrolyte

t [s]	*R* [Ω]	CPE		*C* [F]
		T [F s^{p-1}]	*p* [-]	
0	1036.83	7.89016E-09	0.977745	6.04356E-09
60	549.566	4.15599E-08	0.876294	9.19313E-09
120	543.27	4.86906E-08	0.867551	9.74075E-09
180	526.584	4.86419E-08	0.867823	9.72035E-09

1.0×10^{-3} M Na_2SO_4 aq.

t [s]	*R* [Ω]	CPE		C [F]
		T [F s^{p-1}]	*p* [-]	
0	1243.70	2.14553E-08	0.909255	7.50011E-09
60	1109.84	3.36239E-08	0.880842	8.46501E-09
120	1068.36	3.35738E-08	0.881474	8.47760E-09
180	1068.37	3.24564E-08	0.883373	8.36514E-09

1.0×10^{-2} M Na_2SO_4 aq.

t [s]	*R* [Ω]	CPE		*C* [F]
		T [F s^{p-1}]	*p* [-]	
0	1102.84	1.15388E-08	0.949922	6.58216E-09
60	814.54	3.77297E-08	0.876048	8.80327E-09
120	803.95	3.68385E-08	0.878530	8.86386E-09
180	770.51	3.51037E-08	0.881655	8.73070E-09

1.0×10^{-1} M Na_2SO_4 aq.

t [s]	*R* [Ω]	CPE		*C* [F]
		T [F s^{p-1}]	*p* [-]	
0	793.765	1.01476E-08	0.961449	6.52686E-09
60	516.502	5.21263E-08	0.861997	9.77208E-09
120	500.512	5.04133E-08	0.864129	9.66777E-09
180	479.110	5.14814E-08	0.862899	9.66445E-09

1.0×10^0 M Na_2SO_4 aq.

t [s]	R [Ω]	CPE		C [F]
		T [F s^{p-1}]	p [-]	
0	385.157	1.34039E-08	0.951677	7.22384E-09
60	178.440	9.68451E-08	0.843413	1.26442E-08
120	175.274	1.23148E-07	0.831573	1.39767E-08
180	170.712	1.28631E-07	0.828595	1.39862E-08

Scheme S1

Glass container

↓ ←Surfactant (BL-4.2 / BL-4SY) and Pure water (and electrolyte)

Mix the surfactant and pure water
(2000 rpm, 3 min and 2200 rpm, 1 min by rotating and revolving mixer)

↓

Leave to stand until all bubbles disappear

↓

Divide the sample and set it in the measuring devices

Rheo-SALS measurement
(Viscosity and scattering,
20 or 60 s interval)

Rheo-impedance measurement
(Viscosity and impedance, 1 kHz-500 kHz)

↓

Analyze the correlation between viscosity, structure (SALS),and electrical properties (impedance) during phase transition
